# Fibroblast growth factor 21 attenuates salt-sensitive hypertension-induced nephropathy through anti-inflammation and anti-oxidation mechanism

**DOI:** 10.1186/s10020-021-00408-x

**Published:** 2021-11-13

**Authors:** Hua-Chun Weng, Xin-Yu Lu, Yu-Peng Xu, Yi-Hong Wang, Dan Wang, Yi-Ling Feng, Zhang Chi, Xiao-Qing Yan, Chao-Sheng Lu, Hong-Wei Wang

**Affiliations:** 1 The College of Medical Technology, Shanghai University of Medicine & Health Sciences, Shanghai, 200000 China; 2 Department of Pediatrics, The First Affiliated Hospital of Wenzhou Medical University, 322 Nanbaixiang Street, Wenzhou, 325000 Zhejiang China; 3 The First Clinical Medical College of Wenzhou Medical University, Wenzhou, 325000 China; 4 Ruian Center of Chinese-American Research Institute for Diabetic Complications, The Third Affiliated Hospital of Wenzhou Medical University, Wenzhou, 325000 China; 5 Key Laboratory of Diagnosis and Treatment of Severe Hepato-Pancreatic Diseases of Zhejiang Province, The First Affiliated Hospital of Wenzhou Medical University, 322 Nanbaixiang Street, Wenzhou, 325000 Zhejiang China

**Keywords:** Hypertension, Renal injury, Fibroblast growth factor 21, AMPK

## Abstract

**Background:**

Patients with salt-sensitive hypertension are often accompanied with severe renal damage and accelerate to end-stage renal disease, which currently lacks effective treatment. Fibroblast growth factor 21 (FGF21) has been shown to suppress nephropathy in both type 1 and type 2 diabetes mice. Here, we aimed to investigate the therapeutic effect of FGF21 in salt-sensitive hypertension-induced nephropathy.

**Methods:**

Changes of FGF21 expression in deoxycorticosterone acetate (DOCA)-salt-induced hypertensive mice were detected. The influence of FGF21 knockout in mice on DOCA-salt-induced nephropathy were determined. Recombinant human FGF21 (rhFGF21) was intraperitoneally injected into DOCA-salt-induced nephropathy mice, and then the inflammatory factors, oxidative stress levels and kidney injury-related indicators were observed. In vitro, human renal tubular epithelial cells (HK-2) were challenged by palmitate acid (PA) with or without FGF21, and then changes in inflammation and oxidative stress indicators were tested.

**Results:**

We observed significant elevation in circulating levels and renal expression of FGF21 in DOCA-salt-induced hypertensive mice. We found that deletion of FGF21 in mice aggravated DOCA-salt-induced nephropathy. Supplementation with rhFGF21 reversed DOCA-salt-induced kidney injury. Mechanically, rhFGF21 induced AMPK activation in DOCA-salt-treated mice and PA-stimulated HK-2 cells, which inhibited NF-κB-regulated inflammation and Nrf2-mediated oxidative stress and thus, is important for rhFGF21 protection against DOCA-salt-induced nephropathy.

**Conclusion:**

These findings indicated that rhFGF21 could be a promising pharmacological strategy for the treatment of salt-sensitive hypertension-induced nephropathy.

## Introduction

Hypertension is an imbalance in the regulation of normal blood pressure caused by the interaction of polygenetic inheritance and multiple environmental factors, which has become a serious public health problem affecting 30% of adults around the world (Fatani et al. 2021; Yamamoto-Hanada et al. 2021). High salt intake is one of the important environmental factors, which is closely related to the occurrence and development of hypertension (Hirohama and Fujita 2019). Elevated blood pressure with relatively high salt intake is defined as salt-sensitive hypertension (SSHT) (Abais-Battad et al. 2019). SSHT is the most common type of hypertension in clinic. About 60% of patients with hypertension are sensitive to salt accompanied with uncontrollable blood pressure and serious complications (Rust and Ekmekcioglu 2017). Hypertension is the main risk factor for chronic kidney disease and one of the most common causes of renal failure (Gargiulo et al. 2015). To date, the treatment of hypertensive nephropathy is limited, such as controlling blood pressure and regulating living habits. Once patients develop end-stage renal disease, undergoing dialysis and kidney transplantation are only ways (Hart and Bakris 2010). Furthermore, studies have shown that patients with salt-sensitive hypertension tend to have more severe kidney damage and a higher rate of development of end-stage kidney disease (Slagman et al. 2012). However, the molecular mechanism of the etiology of salt-sensitive hypertensive nephropathy (SSHN) is not clear and no effective treatment has been developed.

Fibroblast growth factor 21 (FGF21), a member of the FGF superfamily, is widely expressed in liver, adipose tissue, pancreas and other metabolic organs and plays metabolic regulatory roles in form of endocrine (Tezze et al. 2019). FGF21 has long been considered as a secretory factor regulating glucose and lipid metabolism (Geng et al. 2020). Previous studies have shown that administration of recombinant FGF21 reduces the levels of blood glucose and lipids and improves insulin sensitivity in type 2 diabetic mice (Ye et al. 2015). Moreover, it is worth noting that some clinical studies have found a significant increase in circulating FGF21 levels in patients with various kidney diseases, including renal transplantation, early diabetic nephropathy, acute renal insufficiency and long-term peritoneal dialysis (Salgado et al. 2021). Strong evidences indicate that FGF21 treatment in vivo effectively reduces renal structure and function damage caused by type 1 and type 2 diabetes, improves renal tubular epithelial cells injury, and significantly decreases the expression of inflammatory reaction and oxidative damage factors (Shao et al. 2015; Cheng et al. 2016). Of note, in Angiotensin II-induced hypertensive mouse model, FGF21 increases angiotensin-converting enzyme 2, which in turn converts angiotensin II to angiotensin-(1–7), then reduces blood pressure and improves vascular inflammation, fibrosis and oxidative stress (Lin and Hardie 2018). Several animal studies have also revealed that FGF21 deficiency aggravates the severity of diabetic nephropathy and atherosclerosis in mice, suggesting the potential organ protective role of FGF21 in chronic metabolic diseases (Lin et al. 2015; Zhang et al. 2018). However, the role of FGF21 in SSHN and its underlying molecular mechanism has never been explored.

Adenosine monophosphate-activated protein kinase (AMPK) is a key molecule of energy metabolism in organisms (Carling 2017). The activation of AMPK depends on the phosphorylation of Thr172 of α subunit (Pan et al. 2018). AMPK plays an important role in glucose and lipid metabolism, cell survival and inflammation (Yan et al. 2018b). Recent studies have found that AMPK activation significantly reduces the expression of inflammatory mediators and improves tissue damage caused by oxidative stress (Hu et al. 2020; Yu et al. 2020). The anti-inflammatory effect of AMPK is mainly through the activation of Sirtuin1 (Sirt1), which directly deacetylates the p65 subunit of NF-κB complex and inhibits the transactivation capacity of p65 subunit and then down-regulates the transcription of NF-κB-dependent inflammation-related genes(Kauppinen et al. 2013; Zheng et al. 2020). In addition, it has reported that AMPK activation promotes the entry of nuclear factor erythroid 2-related factor-2 (Nrf2) into the nucleus, up-regulates the expressions of antioxidant factors such as heme oxygenase-1 (HO-1) and NAD (P) H:quinone oxidoreductase (NQO-1), thus exerting the effect of antioxidant stress (Ni et al. 2019; Yamakage et al. 2020). Notably, the injury of renal tubular epithelial cells induced by releasing of inflammatory mediators and oxidative stress plays a key role in the pathogenesis of hypertensive nephropathy (Ding et al. 2019; Zhang et al. 2020). Moreover, a study shown that FGF21 prevented type 2 diabetic-induced cardiomyopathy through activation of AMPK-mediated antioxidation and lipid-lowering effects (Yang et al. 2018). Another reported that FGF21 promotes ischaemic angiogenesis under diabetic condition was AMPK dependence (Dai et al. 2021). Nonetheless, whether FGF21 can antagonize the inflammatory response and oxidative stress associated with SSHN through AMPK activation remains to be determined.

## Materials and methods

### Animals

Male C57BL/6J mice (wild-type, WT), 8 weeks old, (18–22 g of body weight), were purchased from the Experimental Animal Center of Beijing University of Medical Science (Beijing, China). FGF21 knock-out mice (FGF21-KO) on a C57BL/6J background (18–22 of body weight) were given as a gift from Dr. Steve Kliewer, University of Texas Southwestern Medical Center. These homozygous FGF21-KO mice were backcrossed with C57BL/6J WT mice. Heterozygous offspring were then further bred to gain WT and FGF21-KO littermates. Genotype of FGF21-KO mice were identified by PCR. Before the experiments, all mice were allowed to acclimate to laboratory for 2 weeks, housed at 20 °C with 12:12-h light–dark cycles and 50–60% humidity. Rodent feeds and tap water were supplied by Experimental Animal Center of Wenzhou Medical University. All procedures were carried out in strict accordance with the Laboratory Animal Guide of Wenzhou Medical University and approved by the Animal Experimental Ethical Inspection of Laboratory Animal Centre of Wenzhou Medical University.

### Establishment of deoxycorticosterone acetate (DOCA)-salt hypertension mouse model and group allocation

We made minor modifications to the operation based on the previously described DOCA-salt mouse model (Weng et al. 2014). Making a flank incision to expose the left kidney, ligated and removed it, then the incision was sutured. After 1 week of recovery, making another incision on the right side to percutaneous implantation a 21-day-release DOCA pellet containing 75 mg of DOCA (Innovative Research of America, Sarasota, FL) under mild ether anesthesia. Then put the mouse in a warm cage. All mice were provided 1% NaCl diet 3 days before DOCA treatment. Subsequently, the mice were divided into five subgroups: Wild type control (WT-Con) group (n = 12; among them, there were 6 littermates of FGF21KO mice in a separate cage.), FGF21 knock-out control (FGF21KO-Con) group (n = 6), WT plus DOCA treatment (WT-DOCA) group (n = 12; among them, there were 6 littermates of FGF21KO mice in a separate cage.), FGF21-KO plus DOCA treatment (FGF21KO-DOCA) group (n = 6), WT-DOCA plus rhFGF21 treatment (WT-DOCA-FGF21) group (n = 6). Sham operation was performed in the WT-Con group and FGF21KO-Con group. Salt-sensitive hypertensive nephropathy was induced in WT-DOCA, FGF21KO-DOCA, WT-DOCA-FGF21 group by DOCA-salt treatment as described above. Once the DOCA treatment completed, WT-DOCA-FGF21 group were injected intraperitoneally with rhFGF21 (Provided by the Key Laboratory of Biotechnology Pharmaceutical Engineering, Wenzhou Medical University) at 0.5 mg/kg body weight daily for 8 days, whereas other groups were given the same volume of 0.9% normal saline. Blood pressure was measured on day 0, 2, 4, 6 and 8 by tail-cuff using the telemetricblood pressure system (BP-2010A, Softron Biotechnology, Japan) before the mice were sacrificed, and 24-h urine was collected at 8 days for measurement of urinary albumin and creatinine. All mice were sacrificed 8 days after DOCA-salt treatment and their blood samples and kidney tissues were collected for subsequent studies.

### Measurements for renal function, IL-6, TNF-α and FGF21

Urine albumin, creatinine were measured with an ELISA kit (Exocell; Shibayagi, Gunma, Japan) and Quantichrom™ creatinine assay kit (Bioassay system; CA), respectively according to the manufacturer’s instructions and calculating the albumin-to-creatinine ratio (ACR) (milligram of albumin per gram of creatinine). IL-6, TNF-α and FGF21 concentrations were determined by specific ELISA kits (Abcam; IL-6: ab222503 for mouse and ab178013 for human; TNF-α: ab208348 for mouse and ab181421 for human; FGF21: ab208348 for mouse) with the manufacturer’s instructions. All samples were repeated and the results were averaged.

### Determinations of superoxide dismutase (SOD) activities and malondialdehyde (MDA) concentrations

The homogenate of serum and kidney tissue was centrifuged at 12,000×*g* at 4 °C for 20 min for the supernatant to analysis. SOD activities and MDA levels were measured by using commercial SOD and MDA kits (A001-3-2 and A003-1-2; Nanjing Jiancheng Bioengineering Institute; Nanjing, China) according to the manufacturer’s instructions. The results were expressed as U/ml and nmol/mg protein, respectively.

### Renal histopathological analysis

Part of the cut kidney tissue was soaked in 4% formaldehyde for 24 h, dehydrated in graded ethanol series, and embedded in paraffin for renal histopathological analysis. The tissue blocks were cut into 4.5-μm-thick sections, dewaxed, and hydrated. Masson trichrome staining (Beijing Solarbio Science & Technology Co., Ltd.) was used to estimate the severity of tubulointerstitial fibrosis according to the manufacturer’s instructions. Hematoxylin–eosin staining was performed to observe the tubulointerstitial injury following the manufacturer’s instructions (Beijing Solarbio Science & Technology Co., Ltd.). Microscopic images were obtained with a × 20 objective using an Olympus BX-51 microscopy (Olympus Corporation, Tokyo, Japan) for analysis of the area of the tubulointerstitial injury and tubulointerstitial fibrosis and the positive areas were quantified with Image-Pro plus 6.0 software (Media Cybemetics, Bethesda, USA).

### Immunohistochemistry analysis

For immunohistochemical staining, the kidney sections (4.5 µm) were incubated with 3% H_2_O_2_ at 37 °C for 10 min to inhibit the endogenous peroxidase activity and boiled in antigen retrieval buffer containing citrate-hydrochloric acid (C8532, Sigma, Missouri, USA) for 15 min. Subsequently, the sections were blocked with 5% normal goat serum (OriGENE Technologies, Inc.) for 30 min. After washing, the samples were incubated with primary antibodies F4-80 (1:100; Abcam, ab100790), IL-6 (1:200; Abcam, ab208113), HO-1(1:200; Abcam, ab52947) and NOQ-1 (1:200; Abcam, ab227520) overnight at 4 °C. The slices were then incubated with secondary antibody (1:200; A0277; Beyotime, Shanghai, China; Goat anti-rabbit IgG-HRP), visualized with diaminobenzidine (brown color; OriGene Technologies, Inc.). Images of representative tissue spots were captured with Olympus BX-51 microscope (Olympus Corporation, Tokyo, Japan) and the positive areas were quantified with Image-Pro plus 6.0 software (Media Cybemetics, Bethesda, USA).

### Western blot assay

Kidney tissues were homogenized in RIPA lysis buffer (P0013C, Beyotime, Shanghai, China) and supernatants were collected after centrifugation at 12,000×*g* at 4 °C for 20 min. After tested the total protein concentration by BCA method (P0012S, Beyotime, Shanghai, China), equal amount of protein (30 µg) was separated by SDS-PAGE and transferred to polyvinylidene difluoride (PVDF) membrane. 5% non-fat skimmed milk was used to block membrane at room temperature for 1 h before incubated it overnight at 4 °C with the primary antibodies: FGF21 (1:1000; Abcam, ab171941), HO-1 (1:1000; Abcam, ab52947), NQO-1 (1:1000; Abcam, ab227520), AMPK (1:1000; CST, Cat5832s), P-AMPK (Thr172; 1:1000; CST, Cat8208s), Nrf2 (1:1000, Abcam, ab137550), NF-κB p65 (1:1000, Abcam, ab32536), GAPDH (1:1000, Abcam, ab245355) and Lamin B (1:1000, Abcam, ab16048). All membranes were washed with Tris-buffered saline and 0.1% Tween 20 (TBS-T) three times and incubated with secondary antibody (1:2,000; Santa Cruz Biotechnology; sc-2004; Goat anti-rabbit IgG-HRP) for another 1 h. Finally, the bands were visualized using enhanced chemiluminescence (WP20005, Thermo Fisher Scientific, California, USA) and quantified with Image J software (National Institute of Mental Health, Bethesda, Maryland, USA). Western primary and secondary antibodies removal solution (P0025, Beyotime Institute of Biotechnology) was used for reprobing the PVDF membranes.

### Quantitative real-time PCR (qRT -PCR)

Quantitative real‐time PCR (qRT-PCR) was used to measure the level of FGF21, IL-6, TNF-α mRNA expression. Total RNA was isolated from kidney tissue and HK-2 cells by using a TRIzol Kit (Cat15596026, Invitrogen, Carlsbad, CA, USA) according to the manufacturer’s protocols. In order to the quantify the amount of mRNA, PrimeScript RT reagent kit (KR107, Tiangen Biotech Co., Ltd., Beijing, China) was used to synthesize cDNA from 1 μg of total RNA in a final volume of 20 μg and the 7500 fast real-time PCR system (Applied Biosystems) was used for amplification and detection with SYBR Green kit (FP205, Tiangen Biotech Co., Ltd.) following the manufacture’s guidelines. All expression Ct values of target genes were analyzed by the 2^−ΔΔCT^ methods. The specific primers (Sangon, Shanghai, China) used for the study are listed in Table 1.

**Table 1:** Primers used in this study

Gene	Forward primer (5′–3′)	Reverse primer (5′–3′)
Mouse		
FGF21	AGATCAGGGAGGATGGAACA	TCAAAGTGAGGCGATCCATA
IL-6	TCTATACCACTTACAAGTCGGA	GAATTGCCATTGCACAACTCTTT
TNF-α	CCTGTAGCCCACGTCGTAG	GGGAGTAGACAAGGTACAACCC
GAPDH	TTCCTACCCCCAATGTATACCG	CATGAGGTCCACCACCCTGT
Human		
IL-6	GCCAGAGCTGTGCAGATGAGT	TGGCATTTGTCGTTGGGTCAG
TNF-α	TTCTGCCTGCTGCACTTTGGAG	AGGGCTGATTAGAGAGAGGTCCCTG
GAPDH	AAAATCAAGTGGGGCGATGC	GATGACCCTTTTGGCTCCCC

### Nuclear protein extraction

Nuclear fractions were extracted from fresh kidney tissues by using a nuclear protein extraction kit (P0028, Beyotime, CN) according to the manufacturer's instructions.

### Cell culture

HK-2 cells were purchased from Kunming Cell Bank, Chinese Academy of Sciences. All the cell cultures should be tested for mycoplasma contamination and the results were negative before use. Cells were cultured in DMEM (Gibco; Thermo Fisher Scientific, Inc.) supplemented with 10% FBS (Gibco; Thermo Fisher Scientific, Inc.), 100 U/ml penicilin, and 100 µg/ml streptomycin in a 5% CO_2_ incubator at 37 °C. HK-2 cells were used for in vitro experiments and were divided into four groups as follows: Control group (HK-2 cells were treated with 0.1% DMSO as a negative control) (Dimethyl sulfoxide, DMSO; CLS3085; Sigma, St. Louis, MO, USA), PA group (HK-2 cells induced with 1 mmol/l PA for 24 h) (Palmitate acid, PA; P9767-5G; Sigma, St. Louis, MO, USA), FGF21 group (HK-2 cells were pre-treated with 50 ng/ml rhFGF21 for 2 h, and cultured in the presence of 1 mmol/l PA for 24 h) and FGF21 plus Compound C group (HK-2 cells were pre-treated with 10 µmol/l Compound C and 50 ng/ml rhFGF21 for 2 h, and cultured in the presence of 1 mmol/l PA for 24 h) (HY-13418A; Compound C, a ATP competitive AMPK inhibitor, MCE).

### Statistical analysis

All experiments were independently repeated three times. Data were presented as the mean ± standard deviation. GraphPad Prism 7.0 (GraphPad Software, Inc., La Jolla, CA USA) was used to analyze the results using one-way analysis of variance and Student’s *t*-test. Multiple comparisons between groups were analyzed using Tukey’s post-hoc test. P < 0.05 was considered statistically significant.

## Results

### Circulating levels and renal expression of FGF21 in mice are largely increased in DOCA-salt-induced hypertensive renal damage

**Fig. 1:**
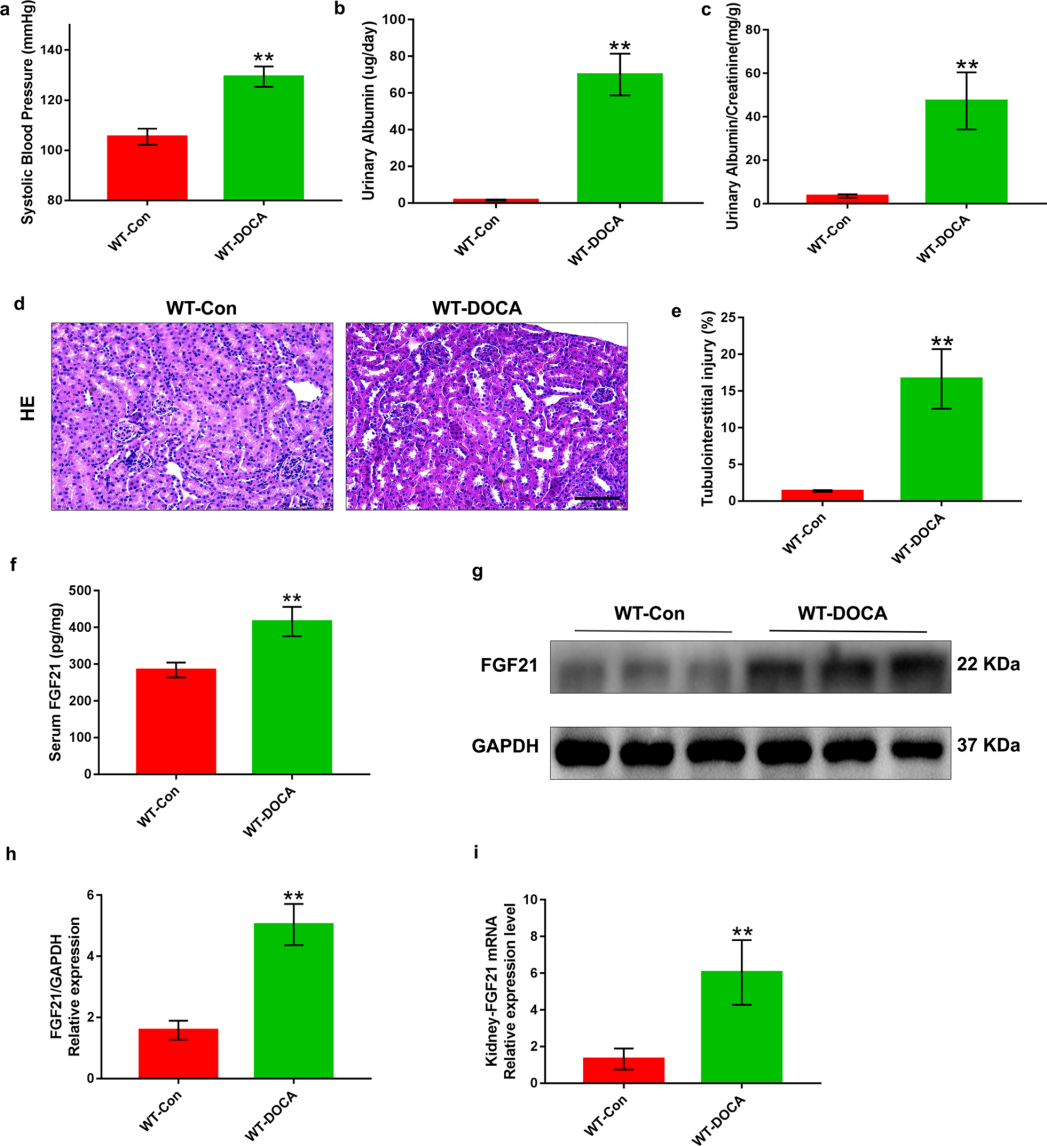
Treated with DOCA-salt for 8 days induced SSHN and increased expression of FGF21 in mice. Eight-week-old male FGF21 wild type (WT) mice based on C57BL/6J were treated with DOCA-salt or sham operation as controls.** a** The systolic blood pressure was monitored by a tail-cuff in DOCA-salt-induced hypertensive mice. **b** and** c** Urinary albumin levels and urinary albumin/creatinine ratio were elevated by treatment with DOCA-salt for 8 days. **d** and **e** Representative images of Hematoxylin–eosin-stained kidney sections showed obvious tubulointerstitial injury evidenced by aggravated epithelial cell necrosis and interstitial oedema in DOCA-salt-treated mice compared with that in the controls.** f** Serum FGF21 levels in the control and DOCA-salt-treated mice were determined by ELISA. **g** and** h** The changes of renal FGF21 expression in mice after treated with DOCA-salt for 8d using Western blot. GAPDH was used as loading and normalization control. **i** FGF21 mRNA expression in kidney tissues for the control and DOCA-salt-treated mice by Q-PCR analysis and values normalized to GAPDH. Quantitative data are presented as mean ± SD, n = 6 mice/group. *P < 0.05, **P < 0.01 versus the control group. Scale bar: 100 μm

To determine the successful establishment of a mouse model of salt-sensitive hypertensive nephropathy, the blood pressure, urinary albumin and urinary albumin/creatinine ratio were preliminarily evaluated. The DOCA-salt treatment for 8 days in mice significantly increased blood pressure and led to high levels of urinary albumin and urinary albumin/creatinine ratio when compared to control mice (Fig. 1a–c). Accordingly, the results of hematoxylin–eosin staining indicated a significant increase in renal tubular lesion in DOCA-salt-treated mice compared with that in the controls (Fig. 1d, e). These results established the mouse model of hypertensive renal damage induced by DOCA-salt treatment for 8 days. To explore the relationship between FGF21 and salt-sensitive hypertensive nephropathy, we investigated the changes of circulating FGF21 levels and its renal expression after treated with DOCA-salt for 8 days in mice. Interestingly, serum FGF21 level was markedly increased accompanied with a dramatic increase in both protein and messenger RNA (mRNA) expression of FGF21 in the kidney of mice (Fig. 1f–i). Taken together, these results suggest that up-regulated circulating levels and renal expression of FGF21 are associated with DOCA-salt-induced hypertensive renal damage and FGF21 may be related to the pathogenesis of salt-sensitive hypertensive nephropathy.

### FGF21 deficiency aggravates DOCA-salt-induced hypertensive renal damage

**Fig. 2:**
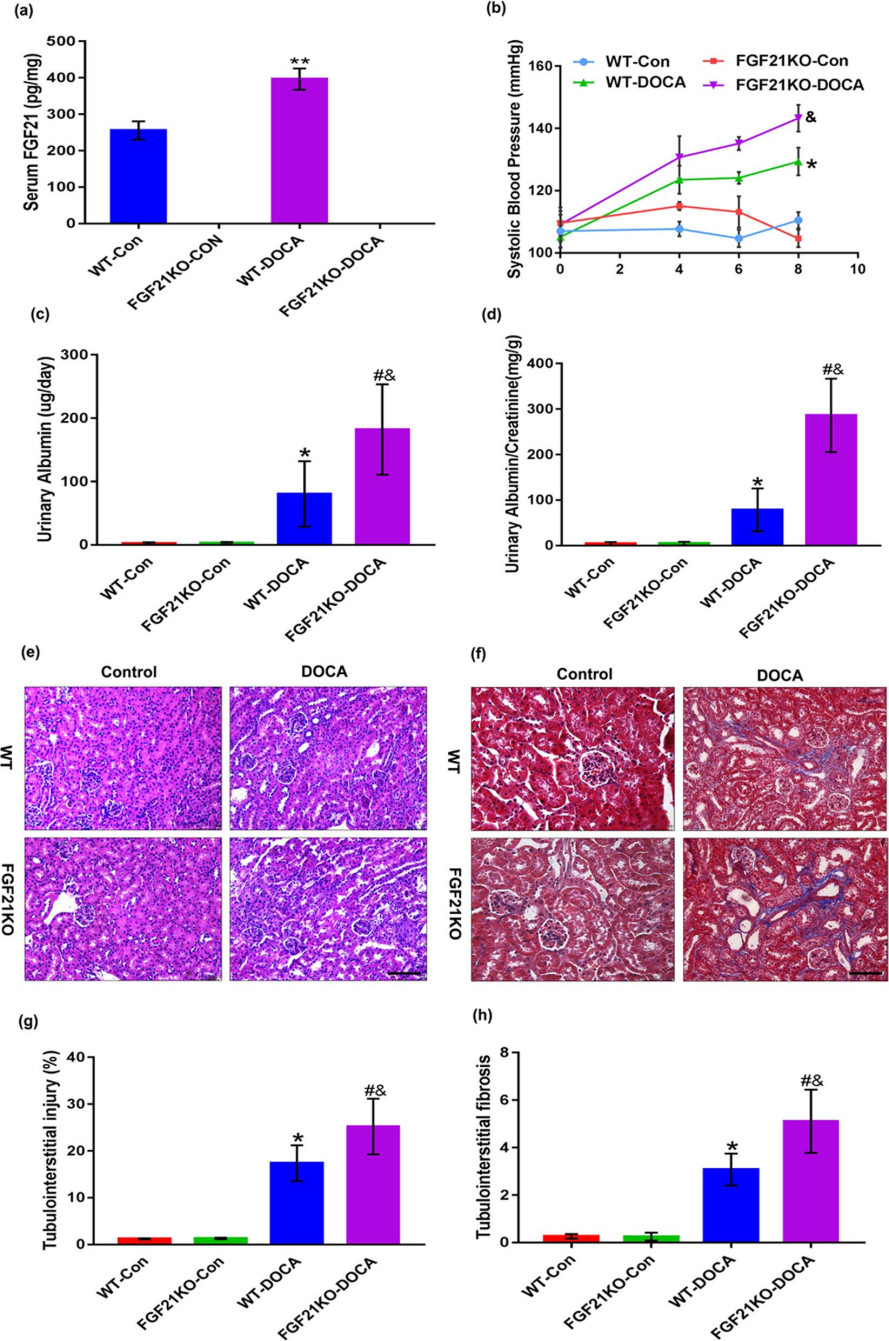
FGF21 deficiency enhanced DOCA-salt-induced hypertensive renal damage in mice. Eight-week-old male FGF21 wild type (WT) and FGF21 knockout (KO) mice based on C57BL/6J were treated with DOCA-salt or sham operation as controls. **a** Serum FGF21 levels in each group were determined by ELISA. **b** Changes in systolic blood pressure measured by a tail-cuff within 8 days at various time points. **c** and** d** Urinary albumin levels and urinary albumin/creatinine ratio were monitored at 8 days in various group mice. **e** and** f** Representative images of Hematoxylin–eosin staining and Masson’s trichrome staining in kidney sections harvested at 8d post-DOCA-salt treatment. **g** Quantification of tubulointerstitial injury region from the Hematoxylin–eosin stained images using Image J software. **h** Quantification of fibrotic degree in tubulointerstitium from the Masson's trichrome stained images using Image-Pro plus 6.0 software. The values are expressed as the mean ± SD, n = 6 mice/group. *P < 0.05 and **P < 0.01 versus the WT control group; ^#^P < 0.05 versus the FGF21KO control group; ^&^P < 0.05 versus the WT-DOCA group. Scale bar: 100 μm

It is worth exploring whether FGF21 is involved in the pathological development of salt-sensitive hypertensive nephropathy. Thus, we made serological and histological comparison between WT and FGF21 KO mice treated with same does of DOCA-salt for 8 days. The circulating levels of FGF21 were detected to confirm the successful establishment of FGF21 KO mice (Fig. 2a). On DOCA-salt treatment, the blood pressure of WT mice increased substantially as expected; however, in FGF21 KO mice the rising amplitude of blood pressure was much higher after DOCA-salt treatment, indicating that FGF21 may be involved in the regulation of blood pressure (Fig. 2b). Moreover, DOCA-salt induced elevations of urinary albumin and urinary albumin/creatinine ratio were dramatically augmented in FGF21 KO mice when compared with WT mice (Fig. 2c, d). Next, we further performed hematoxylin–eosin staining and Masson staining to evaluate the effect of FGF21 deletion on renal pathological damage treated with DOCA-salt and confirmed that the extent of DOCA-salt-induced tubule injury and tubulointerstitial fibrosis in FGF21 KO mice were much severe when compared to those in WT controls (Fig. 2e–h). Collectively, these results indicate that FGF21 deficiency aggravates DOCA-salt-induced hypertensive renal damage in mice.

### Replenishment of rhFGF21 ameliorates salt-sensitive hypertension-induced renal injury in mice

**Fig. 3:**
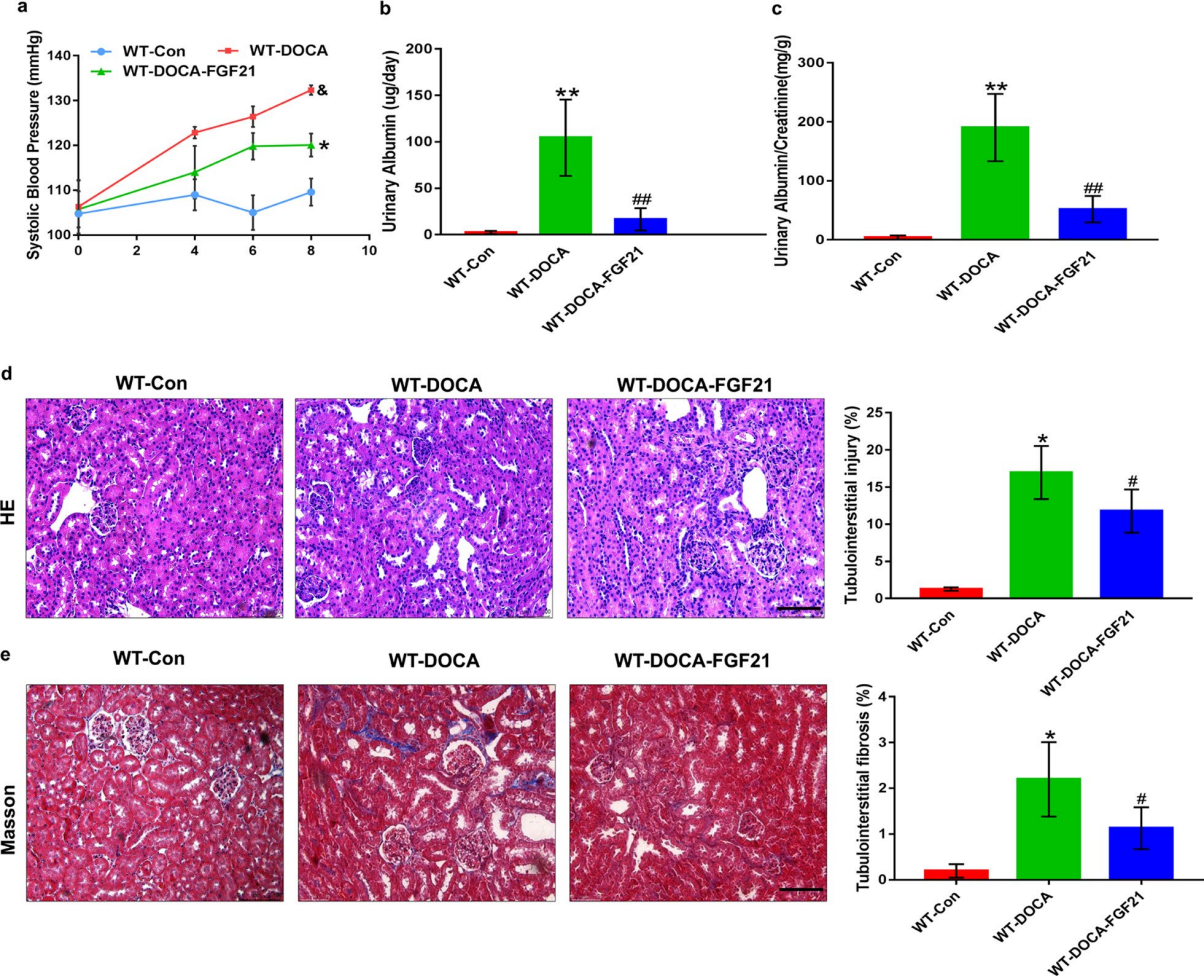
Replenishment of rhFGF21 effectively protected mice from DOCA-salt-induced renal injury. Eight-week-old male WT mice were randomly divided into three experimental groups: the WT-Control group treated with sham operation, followed by intraperitoneal injection with 0.9% normal saline; the WT-DOCA group treated with DOCA-salt; the WT-DOCA-FGF21 group treated with DOCA-salt, followed by intraperitoneal supplementation of recombinant human FGF21 (rhFGF21, 500 μg/kg/day body weight). After 8 days of intervention, the serum and kidney tissues of mice in each group were harvested for detection. **a** Systolic blood pressure (mm Hg) was determined at the indicated days (from 0 to 8 day). **b** and** c** Urinary albumin (μg/day) and the ratio of urinary albumin/creatinine (mg/g) as indexes of renal function were determined. **d** Representative images of Hematoxylin–eosin staining in kidney tissues collected at 8 days post-DOCA-salt treatment and quantification of tubulointerstitial injury region using Image-Pro plus 6.0 software. **e** The levels of fibrosis in kidney tissues harvested at 8 days post-DOCA-salt treatment was determined by Masson's trichrome staining and quantification of fibrotic degree in tubulointerstitium using Image-Pro plus 6.0 software. The values are expressed as the mean ± SD, n = 6 mice/group. *P < 0.05 and **P < 0.01versus the WT control group; ^#^P < 0.05 and ^##^P < 0.01 versus the WT-DOCA group. Scale bar: 100 μm

Given the exacerbation of renal injury induced by DOCA-salt in FGF21-deficient mice, we sought to determine whether replenishment of FGF21 could effectively treat or prevent salt-sensitive hypertensive nephropathy in mice. Not surprisingly, the blood pressure of WT mice was gradually elevated after treated with DOCA-salt for 8 days, as accompanied by increasing urinary albumin and urinary albumin/creatinine ratio (Fig. 3a–c). In addition, extensive renal tissue damage including tubule injury and tubulointerstitial fibrosis were observed in WT mice 8 days after DOCA-salt treatment (Fig. 3d, e). Yet, these negative effects were significantly ameliorated by intraperitoneal injection with recombinant human FGF21 (rhFGF21, 500 μg/kg/day body weight). Interestingly, treatment with rhFGF21 significantly decreased the rising amplitude of blood pressure (Fig. 3a), confirming that rhFGF21 has an effective blood pressure lowering effect. Furthermore, administration of rhFGF21 strongly protected WT mice from DOCA-salt-induced renal injury, as evidenced by obviously reduced elevation in urinary albumin and urinary albumin/creatinine ratio as well as a marked alleviation in tubule injury and tubulointerstitial fibrosis (Fig. 3b–e). All above results suggest that supplementation of rhFGF21 attenuates salt-sensitive hypertension-induced renal injury in mice.

### The marked inhibitory effect of rhFGF21 supplementation on DOCA-salt-induced renal inflammatory response

**Fig. 4:**
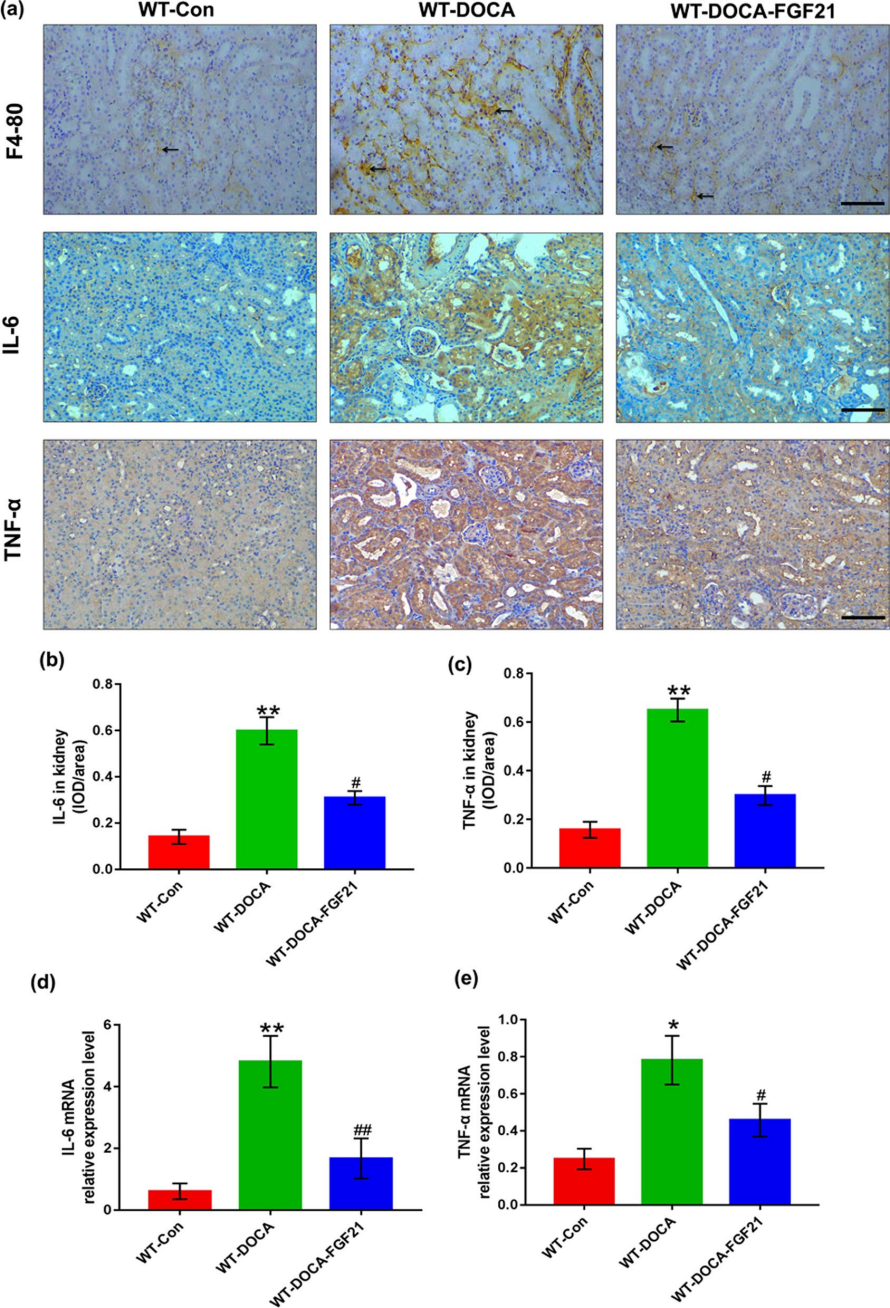
rhFGF21 inhibited DOCA-salt-induced renal inflammatory response in mice. Eight-week-old male FGF21 WT mice were randomly divided into three experimental groups: the WT-Control group treated with sham operation, followed by intraperitoneal injection with 0.9% normal saline; the WT-DOCA group treated with DOCA-salt; the WT-DOCA-FGF21 group treated with DOCA-salt, followed by intraperitoneal supplementation of recombinant human FGF21 (rhFGF21, 500 μg/kg/day body weight). Kidney tissues were harvested at 8d after intervention. **a** Representative images of kidney sections stained with anti-F4-80 antibody, anti-IL-6 antibody and anti-TNF-α antibody, respectively, harvested from mice at 8 days after receiving DOCA-salt treatment. The arrow indicates F4/80 positive cells. **b** and** c** Quantifications of IL-6 and TNF-α protein expression in the kidney tissues of each group were determined by using the mean integrated optical density (IOD)/area with Image-Pro plus 6.0 image analysis software. **d** and** e** Kidney mRNA expression levels of IL-6 and TNF-α were determined by Q-PCR and values normalized to GAPDH. Data are presented as Mean ± SD, n = 6 mice/group. *P < 0.05, **P < 0.01 versus the WT control group; ^#^P < 0.05, ^##^P < 0.01 versus the WT-DOCA group. Scale bar: 100 μm

To explain the molecular basis of rhFGF21 improving DOCA-salt-induced hypertensive renal injury, we detected the degree of renal inflammation in each group of mice with or without rhFGF21 treatment. We determined whether rhFGF21 supplementation prevents the infiltration of macrophages in the renal tubulointerstitium. Under the treatment of DOCA-salt for 8 days, a large number of F4/80 (a murine macrophage maker) positive cells migrated into the renal tubulointerstitium which may be associated with severe injury of renal tubular epithelial cells. However, replenishment of rhFGF21 reduced the number of infiltrating F4/80 positive cells (Fig. 4a). We next determined whether rhFGF21 supplementation reduces the release of TNF-α and IL-6 with immunohistochemical staining and Q-PCR. The results revealed that replenishment of rhFGF21 significantly decreased DOCA-salt-induced renal inflammatory factors accumulation at both protein and mRNA level (Fig. 4a–e). These results suggest that rhFGF21 treatment protects against DOCA-salt-induced hypertensive renal injury by inhibiting the inflammatory response in the kidney.

### rhFGF21 supplementation restores the antioxidant ability of kidney in DOCA-salt-induced hypertensive renal injury mice

**Fig. 5:**
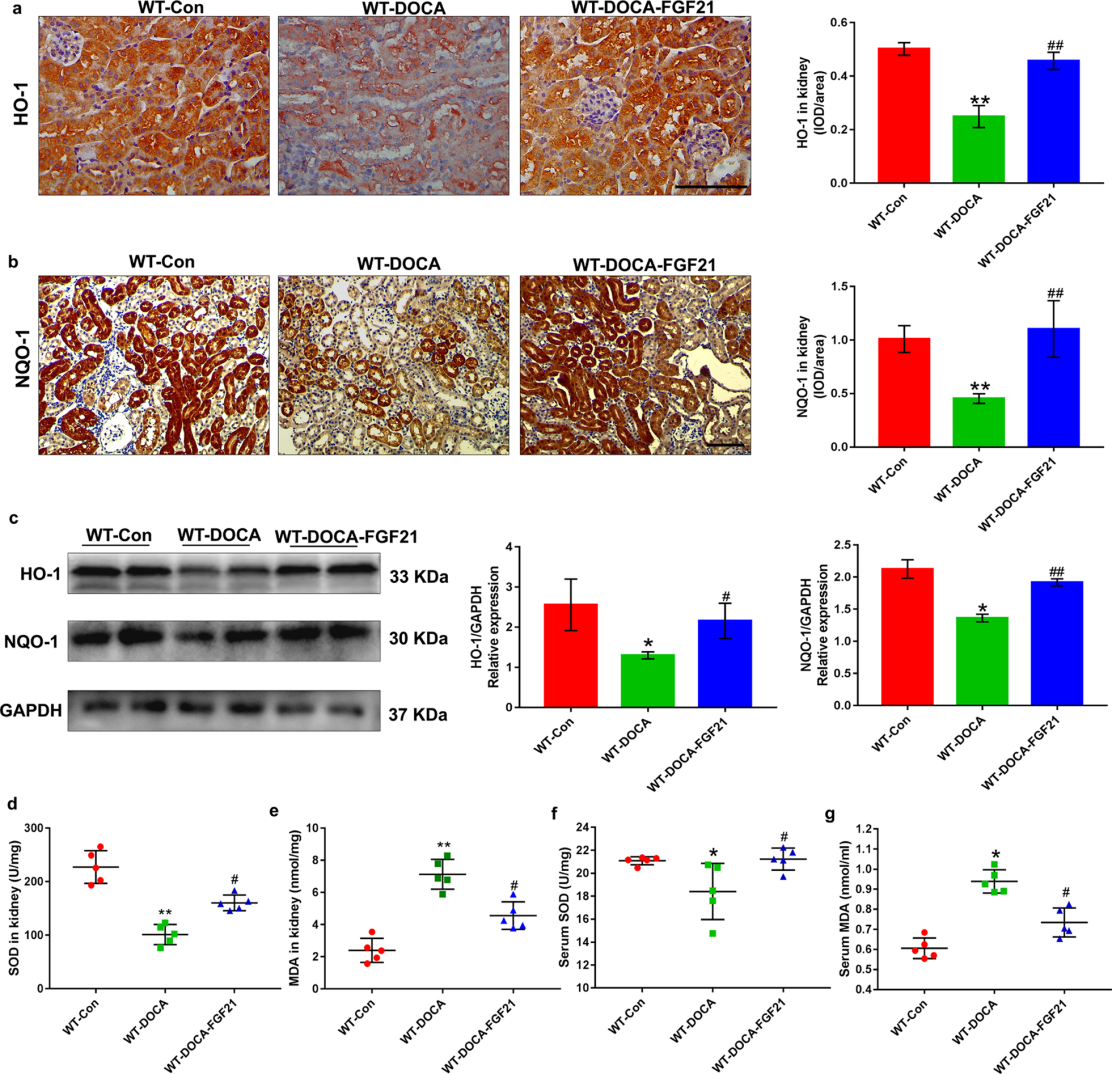
rhFGF21 improves the impaired antioxidant activities of kidney in mice treated with DOCA-salt. Eight-week-old male FGF21 WT mice were randomly divided into three experimental groups: the WT-Control group treated with sham operation, followed by intraperitoneal injection with 0.9% normal saline; the WT-DOCA group treated with DOCA-salt; the WT-DOCA-FGF21 group treated with DOCA-salt, followed by intraperitoneal supplementation of recombinant human FGF21 (rhFGF21, 500 μg/kg/day body weight). Serum and kidney tissues were harvested at 8 days after intervention. **a** and** b** Kidney sections were collected and stained with antibody against HO-1 and NQO-1 and the quantitative mean integrated optical density (IOD)/area of HO-1 and NQO-1 were analyzed by Image-Pro plus 6.0 in each group were shown. **c** Protein expressions of HO-1 and NQO-1 were determined by Western blot. GAPDH was used as loading and normalization control and the quantifications of protein expressions of HO-1 and NQO-1 were analyzed by Image J. **d** and** e** Effects of rhFGF21 on malonyldialdehyde (MDA) and superoxide dismutase (SOD) levels of kidney tissues at 8 days after DOCA-salt treatment. **f** and** g** Serum levels of MDA and SOD were measured at 8d after DOCA-salt treatment in each group. Data are presented as Mean ± SD, n = 5–6 mice/group. *P < 0.05, **P < 0.01 versus the WT control group; ^#^P < 0.05, ^##^P < 0.01 versus the WT-DOCA group. Scale bar: 100 μm

Given the dramatic inhibitory role of rhFGF21 on DOCA-salt-induced renal inflammation, we sought to determine whether rhFGF21 restores the antioxidant activities of kidney after DOCA-salt treatment. Thus, we measured HO-1 and NQO-1 (two important antioxidant enzymes) in the kidney tissues respectively by immunohistochemical staining and Western blot. Eight days DOCA-salt treatment reduced both the protein expression levels of HO-1 and NQO-1. Conversely, rhFGF21 supplementation significantly reversed DOCA-salt induced reduction of HO-1 and NQO-1 protein expression (Fig. 5a–c). Additionally, to further explore the role of rhFGF21 in antioxidant reactions, we also tested the levels of MDA and SOD, the oxidative stress-related indicators, both in serum and kidney tissues of mice. After DOCA-salt treatment, detection results of serum and renal tissues showed that the concentrations of MDA were significantly elevated, whereas rhFGF21 replenishment was associated with a marked reduced MDA levels compared with WT-DOCA group (Fig. 5e, g). Nonetheless, serum and renal SOD concentrations were significantly decreased within 8 days after DOCA-salt treatment. On the other hand, DOCA-salt-induced reductions of SOD concentrations both in serum and renal tissues were significantly augmented when treated with rhFGF21 compared to those in WT-DOCA group (Fig. 5d, f). These observations indicate that rhFGF21 supplementation restores the antioxidant ability of kidney in DOCA-salt-induced hypertensive renal injury mice.

### rhFGF21 treatment upregulates renal expression of phosphorylated AMPK and mediates the changes of nuclear accumulation of NF-κB p65 and Nrf2 in DOCA-salt-induced hypertensive renal injury mice

**Fig. 6:**
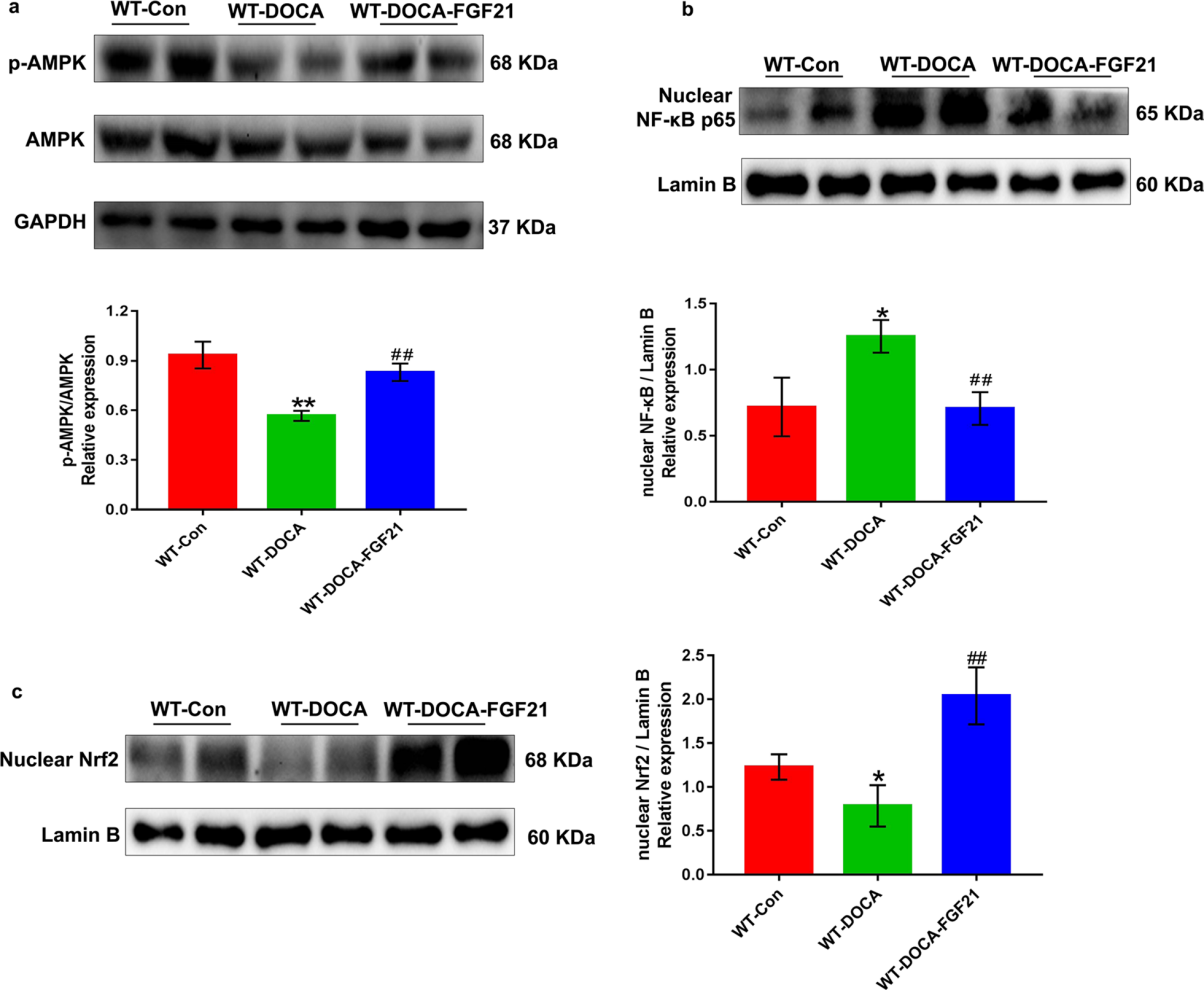
rhFGF21 activated renal AMPK and changed nuclear abundance of NF-κB p65 and Nrf2 in SSHN mice. Eight-week-old male FGF21 WT mice were treated with DOCA-salt or sham operation as controls, followed by intraperitoneal administration of recombinant human FGF21 (rhFGF21, 500 μg/kg/day body weight) or same volume of 0.9% normal saline. Kidney tissues were harvested at 8 days after intervention. **a** The protein expression levels of AMPK and phosphorylated AMPK (p-AMPK) were measured by Western blot using anti-AMPK and anti-p-AMPK antibodies. GAPDH was used as the internal control and the relative ratio of p-AMPK/AMPK was quantified and the normalized values were indicated in the histogram. **b** Western blot results for protein levels of NF-κB p65 in nuclear fractions in the kidney tissues of each group and statistical analysis of the nuclear abundance of NF-κB p65. Lamin B was used as the internal control. **c** The levels of nuclear accumulation of Nrf2 in kidney tissues of each group were detected by Western blot analysis and quantification of the nuclear protein expression of Nrf2 in nuclear fractions was analyzed by Image J. Lamin B was used as loading and normalization control. Data are presented as Mean ± SD, n = 6 mice/group. *P < 0.05, **P < 0.01 versus the WT control group; ^#^P < 0.05, ^##^P < 0.01 versus the WT-DOCA group

We performed Western blot to explore the effects of rhFGF21 on the expression of phosphorylated AMPK as well as the nuclear translocation of NF-κB p65 and Nrf2 in renal tissues of DOCA-salt-induced renal injury mice. The ratio of p-AMPK/AMPK markedly down-regulated after 8 days DOCA-salt treatment. Yet, replenishment of rhFGF21 into DOCA-salt-treated mice resulted in a dramatically elevation of this ratio (Fig. 6a). Likewise, we next investigated whether rhFGF21 supplementation is sufficient to alter the nuclear accumulation of NF-κB p65 and Nrf2 in mouse kidney tissues after treated with DOCA-salt, which could well explain the effective anti-inflammatory and antioxidant stress roles of rhFGF21 on DOCA-salt-induced hypertensive renal injury. Surprisingly, the nuclear translocation of NF-κB p65 was significantly increased followed by DOCA-salt treatment for 8 days, whereas rhFGF21 supplementation reduced the nuclear translocation of NF-κB p65 (Fig. 6b). Conversely, DOCA-salt treatment decreased the nuclear accumulation of Nrf2, while rhFGF21 supplementation significantly reversed this phenomenon (Fig. 6c). These results suggest that rhFGF21 markedly alters the phosphorylation of AMPK and mediates the nuclear accumulation of NF-κB p65 and Nrf2 in DOCA-salt-treated kidney tissues. Thus, we speculate that activation of AMPK may mediate an essential role in the protective roles of rhFGF21 for anti-inflammatory and antioxidant stress.

### Inhibition of phosphorylated AMPK abolishes rhFGF21-induced attenuation of inflammatory response and antioxidant ability in palmitate acid-treated renal tubular epithelial cells

**Fig. 7:**
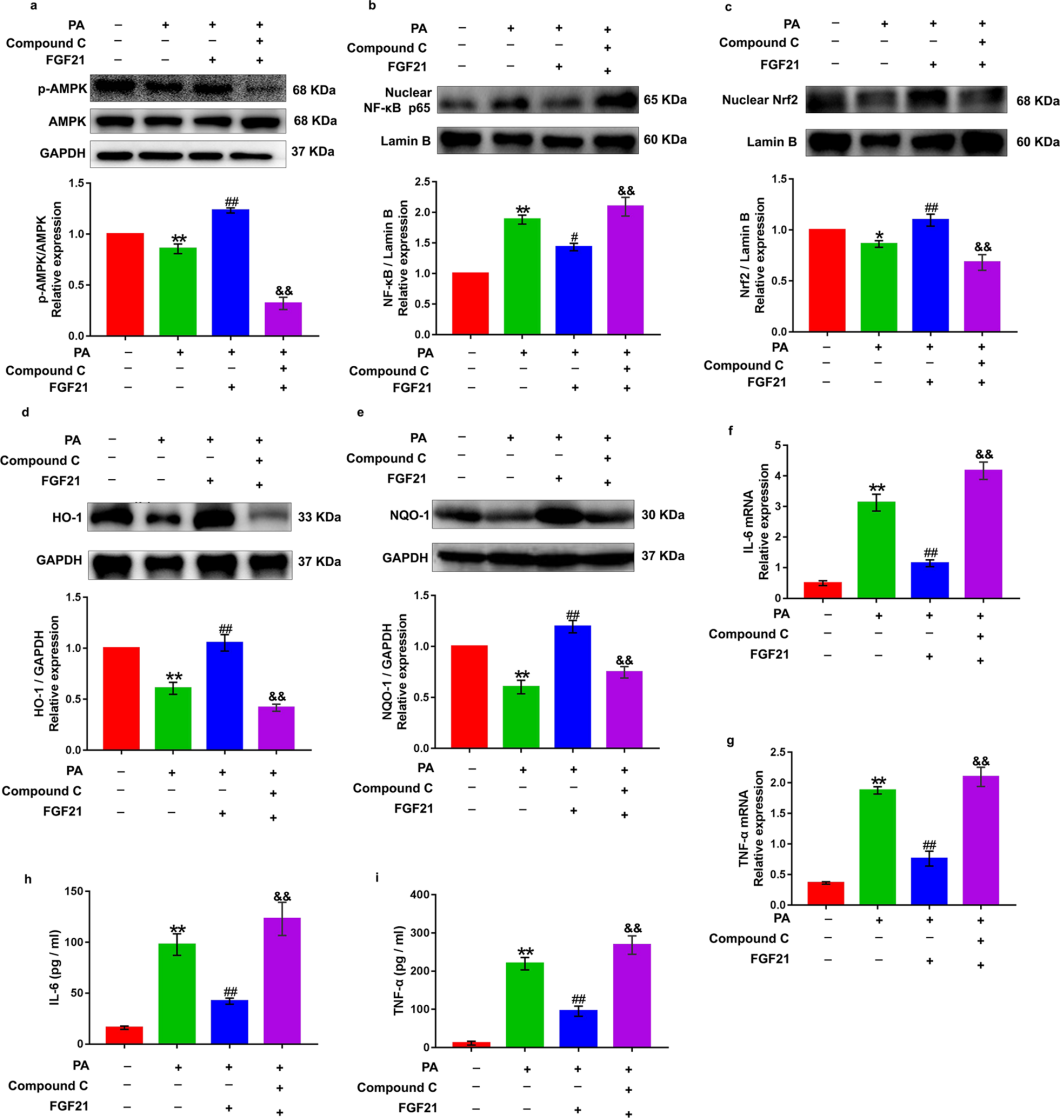
AMPK inhibition blunted rhFGF21-induced attenuation of inflammation and oxidative stress in PA-treated HK-2 cells. Phosphorylated AMPK was inhibited using Compound C (a ATP competitive AMPK inhibitor, 10 μM) in human renal tubular epithelial cells (HK-2), the cells were then pretreated with rhFGF21 (50 ng/ml) for 2 h and subsequently stimulated with overdoes palmitate acid (PA, 1 mM) for 24 h. **a** Western blot analysis showed the effects of rhFGF21 and Compound C pretreatment on PA-induced AMPK phosphorylation in HK-2 cells. Image J was used to quantify the ratio of p-AMPK/AMPK. GAPDH was used as the internal control. **b** and** c** The nuclear translocation levels of NF-κB p65 and Nrf2 in nuclear fractions were detected by Western blot. Quantification of the nuclear protein expression of NF-κB p65 and Nrf2 was analyzed by Image J. Lamin B was used as the internal control. **d** and **e** The expression of antioxidant associated proteins HO-1 and NQO-1 were measured by Western blot. Quantifications of proteins expression of HO-1 and NQO-1 were analyzed by Image J. GAPDH was used as loading and normalization control. **f** and **g** Quantitative RT-PCR determination of IL-6 and TNF-α mRNA and values normalized to GAPDH. **h** and **i** ELISA analysis for the concentrations of IL-6 and TNF-α in HK-2 cells supernatants. The values are expressed as the mean ± SD. *P < 0.05, **P < 0.01 versus the control group; ^#^P < 0.05, ^##^P < 0.01 versus the PA group; ^&^P < 0.05, ^&&^P < 0.01 versus the rhFGF21 group

DOCA-salt treatment induced severe renal tubular epithelial cells injury in mice. Thus, we next determined the inhibitory actions of rhFGF21 in a human renal tubular epithelial cell line, HK-2, stimulated with overdoes palmitate acid (PA, 1 mM) to mimic the hypertensive milieu of inflammatory response and oxidative damage as previously reported (Lu et al. 2020). Moreover, to investigate whether activation of AMPK is responsible for the renal protective effects of rhFGF21 in vitro. We treated HK-2 cells with Compoud C (a ATP competitive AMPK inhibitor, 10 μM) to block the phosphorylation of AMPK, followed by supplementation of rhFGF21 (50 ng/ml) and subsequent PA treatment. Western blot results showed that PA treatment significantly reduced AMPK phosphorylation, whereas treatment of the HK-2 cells with rhFGF21 significantly increased the PA-induced reduction of AMPK phosphorylation, but these two kinds of intervention did not affect the AMPK expression (Fig. 7a). As expected, the up-regulation effect of rhFGF21 on the phosphorylation of AMPK was dramatically blocked by Compound C treatment (Fig. 7a). In addition, overdose PA induced a significant nuclear translocation of NF-κB p65 and resulted in high level synthesis and release of TNF-α and IL-6, which were observed in mRNA levels and supernatant of HK-2 cells using Q-PCR and ELISA (Fig. 7b and f–i). However, pretreatment with rhFGF21 prevented PA-induced nuclear translocation of NF-κB and release of these inflammatory factors (Fig. 7b and f–i). In parallel, we also tested the antioxidant capacity of HK-2 cells in response to PA stimulation with or without rhFGF21 supplementation. Results showed that PA profoundly reduced the nuclear accumulation of Nrf2 and led to significant down-regulation of HO-1 and NQO-1 in HK-2 cells (Fig. 7c–e). However, rhFGF21 supplementation restored the antioxidant activities of HK-2 cells evidenced by significantly increased Nrf2 nuclear accumulation and higher expressions of HO-1 and NQO-1compared to PA treatment (Fig. 7c–e). These data suggest that rhFGF21 has dramatic inhibitory effects on PA-induced inflammatory response and oxidative stress in HK-2 cells. Surprisingly, these anti-inflammatory and antioxidant ability of rhFGF21 in response to PA stimulation in HK-2 cells was prominently weakened by Compound C-induced inhibition of AMPK phosphorylation (Fig. 7b–i). Collectively, our findings indicate that FGF21-induced alteration of nuclear translocation of NF-κB p65 and Nrf2 are mediated by the phosphorylation of AMPK. Besides, the anti-inflammatory and antioxidant ability of rhFGF21 on renal tubular epithelial cells depend on AMPK activation.

## Discussion

In the present study, we noticed that both circulating levels and renal expression of FGF21 were up-regulated in DOCA-salt-induced hypertensive mice. We discovered that the deficiency of FGF21 aggravated DOCA-salt-induced hypertensive nephropathy in mice. Most importantly, we found that replenishment of recombinant human FGF21 (rhFGF21) effectively ameliorates DOCA-salt-induced renal injury in mice. Functionally, we explored that rhFGF21 respectively inhibits and promotes the nuclear translocation of NF-κB p65 and Nrf2 by activating AMPK, thus enhancing the anti-inflammatory and antioxidant ability of the kidney.

We demonstrated that DOCA-salt treatment for 8 days in mice successfully established the mouse model of salt-sensitive hypertensive nephropathy evidenced by markedly elevated blood pressure, urinary albumin, urinary albumin/creatinine ratio as well as severe renal tubular lesion. Besides, DOCA-salt treatment led to a drastic up-regulation in both circulating levels and renal expression of FGF21, suggesting that FGF21 may be involved in the pathogenesis of salt-sensitive hypertensive-induced kidney damage. Increasing animal-based studies have showed that elevated FGF21 expression protects type 1 and type 2 diabetic mice from high glucose-induced renal injury and myocardial hypertrophy (Zhang et al. 2016; Lewis et al. 2019). Meanwhile, researches have also proved that administration of recombinant human FGF21 (rhFGF21) can effectively reduce blood pressure in Angiotensin II-induced hypertensive mouse model (Huang et al. 2017). Furthermore, clinical studies have demonstrated that the levels of circulating FGF21 increased significantly in patients with early diabetic nephropathy and hypertensive nephropathy (Ong et al. 2015; Huang et al. 2017; Lei et al. 2020). Therefore, we speculated that DOCA-salt-evoked increasing expression of FGF21 may represent a stress protection for the kidney to defend against DOCA-salt-induced kidney damage. Herein, we explored two lines of evidence to prove our hypothesis. On the one hand, FGF21 deficiency in mice exhibited significantly elevated blood pressure, exacerbated renal function as well as aggravated renal injury and renal interstitial fibrosis when compared with WT mice in response to DOCA-salt treatment. On the other hand, increased blood pressure and severe kidney damage were markedly reversed by the supplementation of exogenous rhFGF21. Collectively, our data showed that up-regulated renal expression of FGF21 is a compensatory self-protection mechanism for the kidney in response to DOCA-salt stimulation and rhFGF21 may be a promising pharmacological strategy for the treatment of salt-sensitive hypertensive nephropathy.

In the pathogenesis of salt-sensitive hypertension, the uncontrollable hypertensive environment will continue to activate renal inflammation, which is considered to be a central factor for driving the development of salt-sensitive hypertensive nephropathy (Lu and Crowley 2018). Our DOCA-salt-challenged mouse model presented with significantly up-regulated renal expression of pro-inflammatory cytokines including TNF-α and IL-6 and lots of F4/80 positive macrophages infiltrated into the tubulointerstitial region. However, all of which were markedly attenuated by administration of rhFGF21, indicating that the renoprotective effect of FGF21 is pramainly attributed to its ability to inhibit renal inflammatory actions.

NF-κB signaling system is recognized to be a particularly important transcription factor in regulating inflammatory response, which controls the expressions of a host of pro-inflammatory genes (Lawrence 2009). Moreover, complex hypertensive milieu (i.e., hypertension, oxidative stress, glomeruli-leaked free fatty acids, and activated renin-angiotensin system) can activate NF-κB signaling, thereby aggravating renal inflammatory actions and accelerates the progression of hypertensive nephropathy (Yan et al. 2018a; Hirohama and Fujita 2019). Notably, our results confirmed that DOCA-salt treatment evoked the activation of NF-κB in kidney tissue, as evidenced by elevated nuclear translocation of NF-κB p65 and this phenomenon was blocked by rhFGF21 supplementation. Considering that DOCA-salt treatment in mice induced obvious renal tubular epithelial cells damage and tubular epithelial cells are often used to investigate hypertensive nephropathy. In order to further explore whether the anti-inflammatory effect of FGF21 depends on its effective blood pressure-lowering activity. We used overdose PA to stimulate human renal tubular epithelial cell line, HK-2, in vitro to mimic hypertension-like inflammatory response with or without rhFGF21 pretreatment. Interestingly, overdose treatment of HK-2 cells with PA indeed induced severe inflammatory reaction, indicated by activated NF-κB and increased synthesis and release of TNF-α and IL-6; however, all of which were reversed by rhFGF21 replenishment. Overall, our data suggest that both the anti-hypertensive effect of FGF21 and its own anti-inflammatory activity contribute to the inhibition of renal inflammation actions, which is strongly related to the inhibition of NF-κB activation, in the DOCA-salt-induced hypertensive nephropathy mouse model.

In addition to the above-mentioned inflammatory mechanism, renal oxidative stress triggered by DOCA-salt is also an important mediator in the development of salt-sensitive hypertensive nephropathy (Seifi et al. 2010). Hypertensive environment leads to excessive accumulation of peroxynitrite and reactive oxygen species (ROS) in the kidney, which induces gradual consumption of endogenous antioxidants and makes renal cells more sensitive to injury (Sinha and Dabla 2015). The disruption of the balance between oxidation and antioxidation eventually results in renal cells death, thereby aggravating the damage of kidney tissue (Coppolino et al. 2018). In line with these reports, our data showed that DOCA-salt treatment for 8 days induced sharp increase of MDA and significant consumption of endogenous antioxidant enzymes HO-1, NQO-1 and SOD in the kidney tissue of mice, whereas replenishment of rhFGF21 was enough to reverse these changes. Consistent with these result, direct treatment of HK-2 cells with overdose PA in vitro induced marked decrease in the protein expressions of HO-1 and NQO-1, while rhFGF21 effectively restored their protein expressions. Our results suggest that rhFGF21 exerts its antioxidant stress role not only due to its blood pressure-lowering effect, but also rhFGF21 can be recovered as an effective exogenous antioxidant to restore the antioxidant activities of kidney in DOCA-salt-induced hypertensive renal injury mice.

The nuclear factor erythroid 2-related factor-2 (Nrf2), a central transcription factor in activation of the endogenous antioxidant stress defense system through releasing from Keap1 and translocating to the nucleus, has presented superior effect to scavenge DOCA-salt-induced ROS in mice (Gomez-Guzman et al. 2012). Hypertension stimulation reduces the nuclear accumulation of Nrf2 and lead to down-regulation of a cluster of antioxidant genes including HO-1, NQO-1 and GSH-PX, which breaks the kidney redox balance and aggravates renal damage (Choi et al. 2014). In contrast, using Nrf2 inducing compounds confers mice the ability to resist or slow down the progression of hypertensive nephropathy by activating the expression of antioxidant genes (Farooqui et al. 2021). Likewise, our study showed that DOCA-salt treatment decreased the nuclear accumulation of Nrf2 in kidney tissue of mice, which explained the impaired expressions of antioxidant enzymes as previously described; however, these changes were reversed by rhFGF21 supplementation, suggesting that rhFGF21-induced up-regulation of antioxidant enzymes to restore the antioxidant activity of kidney in response to DOCA-salt treatment at least in part mediated by increased nuclear accumulation of Nrf2.

NF-κB and Nrf2 signaling pathways are well-established molecular mechanism for FGF21 to underline its inhibition effects of inflammatory actions and redox imbalance in DOCA-salt-induced hypertensive renal injury mice. Next, we focused on dissecting whether there is a deep-seated common molecular target to explain how FGF21 regulates the nuclear translocation of NF-κB and Nrf2. AMPK is a trimeric serine/threonine protein kinase activated in the form of phosphorylation and considered to be a converter that regulates cell energy metabolism (Gao et al. 2020). Activated AMPK regulates glucose and lipid metabolism to reduce ATP consumption and increase ATP production, meeting cellular energy needs to prevent inflammatory response and oxidative stress caused cell death (Herzig and Shaw 2018). Strong evidence suggests that AMPK-mediated activation of Sirt1 prevents the nuclear-binding ability of NF-κB p65 subunit, which in turn down-regulates the expression of inflammatory cytokines (Kauppinen et al. 2013). In addition, AMPK phosphorylates Nrf2 at serine 374, 408 and 433, moves Nrf2 from the cytoplasm to the nucleus, and binds to the antioxidant response element (ARE) gene to exert its antioxidant effect (Matzinger et al. 2020). There has been demonstrated that FGF21 could restore the nuclear accumulation of Nrf2 by activating AMPK/AKT pathway and enhance the antioxidant function of cardiomyocytes in diabetic cardiomyopathy mice (Yang et al. 2018). Conversely, AMPK deletion inhibits the nuclear accumulation of Nrf2 in type 2 diabetic mice and aggravates the oxidative stress in kidney tissues (Sun et al. 2020). In this study, we demonstrated that not only overdose PA inhibited the phosphorylation of AMPK in renal tubular epithelial cells, but renal tissue from DOCA-salt treated mice presented with down-regulated phosphorylation of AMPK, and rhFGF21 activated AMPK in each of those cases. Although activated AMPK can regulate the nuclear translocation of NF-κB p65 and Nrf2, it is not clear that induction of AMPK by FGF21 is responsible for FGF21-mediated anti-inflammatory and antioxidant ability through NF-κB and Nrf2 signaling pathways. Our observation that the AMPK inhibitor Compound C abrogated the positive effects of rhFGF21 on reduction of nuclear NF-κB p65 abundance, inhibition of inflammatory factor as well as elevation of nuclear Nrf2 accumulation, induction of antioxidant enzymes in PA-stimulated renal tubular epithelial cells. Taken together, our data support the role of AMPK as an essential effector to mediate the anti-inflammatory and antioxidant stress actions of FGF21.

Given the limitations of the current study, certain important issues were not addressed. First of all, our observations are only based on mouse model, we should further determine the role of FGF21 in SSHN in humanoid large animals and in preclinical studies. In addition, we only investigated the anti-inflammatory and antioxidant effects of FGF21 on renal tubular epithelial cells. However, future studies need to determine the role of FGF21 in other kidney cell types. Thirdly, FGF receptors 1–4 are main receptors of FGF21 and it is difficult to determine which receptors plays a major role in the renal protective effects of FGF21. We will further determine the role of FGF21 receptors in the progression of salt-sensitive hypertensive nephropathy in the future. Finally, amounts of free fatty acids (FFA) will be leaked from the glomerulus and absorbed into the proximal renal tubules, resulting in nephrolipotoxicity, and then cause renal tubular epithelial cell necrosis and exfoliation in the progression of hypertensive nephropathy (Liu et al. 2019). FGF21 has a powerful lipid-lowering effect (Byun et al. 2020). Therefore, additional studies are required to determine whether FGF21 can effectively reduce the lipotoxicity of kidney in salt-sensitive hypertensive nephropathy.

## Conclusions

**Fig. 8:**
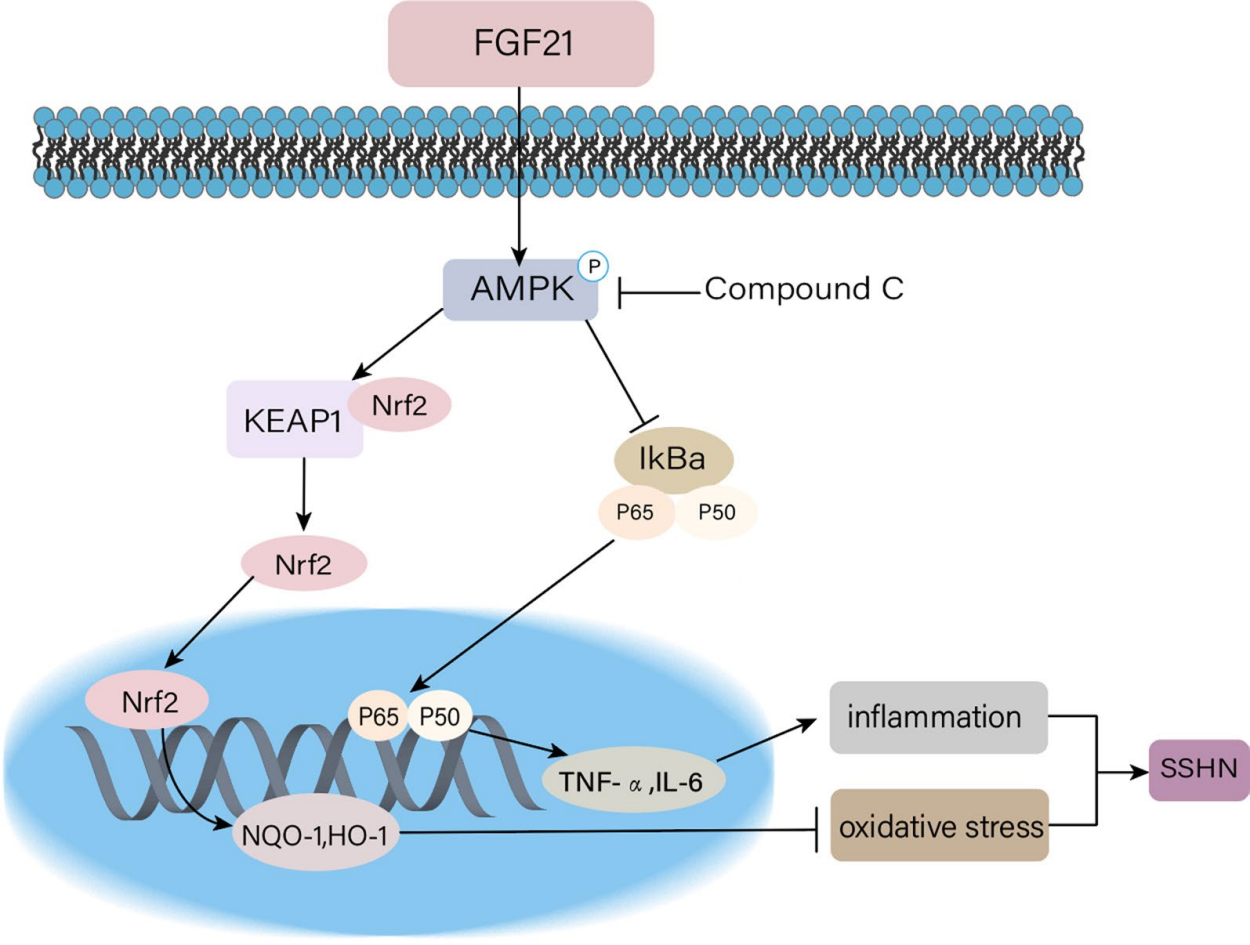
Mechanistic illustration of the basis of FGF21 protection against SSHN. FGF21 inhibits renal inflammation and oxidative stress by promoting AMPK phosphorylation via Nrf2-mediated antioxidant effects and inhibition of NF-κB-related inflammation, resulting in protection against DOCA-salt-induced SSHN in mice

In summary, our findings indicated that the elevation of circulating and renal FGF21 levels is a compensatory stress protection for the kidney in response to DOCA-salt treatment. We demonstrated that rhFGF21 was strongly effective to prevent the progression of DOCA-salt-induced hypertensive nephropathy by inducing AMPK activation, which in turn inhibited NF-κB-mediated inflammatory cytokines release and activated Nrf2-regulated renal antioxidant capacity and thus, is important for FGF21 protection against DOCA-salt-induced hypertensive renal injury (Fig. 8). Our study indicates that the development of novel therapies using FGF21 is promising for future medical interventions of SSHN.

## Data Availability

The data and materials used to support the findings of this study are available from the corresponding author upon request.

## Abbreviations

FGF21: Fibroblast growth factor 21
SSHN: Salt-sensitive hypertensive nephropathy
DOCA: Deoxycorticosterone acetate
rhFGF21: Recombinant human fibroblast growth factor 21
PA: Palmitate acid
Sirt1: Sirtuin1
AMPK: Adenosine monophosphate-activated protein kinase
Nrf2: Nuclear factor erythroid 2-related factor-2
HO-1: Heme oxygenase-1
NQO-1: NAD (P) H:quinone oxidoreductase
NF-κB: Nuclear factor kappa B
SOD: Superoxide dismutase
MDA: Malondialdehyde
FFA: Free fatty acids
CC: Compound C
